# High Endemicity and Low Molecular Diversity of Hepatitis B Virus Infections in Pregnant Women in a Rural District of North Cameroon

**DOI:** 10.1371/journal.pone.0080346

**Published:** 2013-11-12

**Authors:** Alexandra Ducancelle, Pierre Abgueguen, Jacques Birguel, Wael Mansour, Adeline Pivert, Hélène Le Guillou-Guillemette, Jean-Jacques Sobnangou, Amélie Rameau, Jean-Marie Huraux, Françoise Lunel-Fabiani

**Affiliations:** 1 Department of Bacteriology and Virology, Angers University Hospital, and HIFIH Laboratory, UPRES 3859, SFR 4208, Angers Cedex 9, France; 2 Department of Infectious Diseases and Internal Medicine, Angers University Hospital, Angers Cedex 9, France; 3 Catholic hospital of Tokombéré, BP 74 Maroua, Cameroun; 4 Public health district of Tokombéré, BP 115 Mora, Cameroun; 5 Virology Laboratory, Hospital AP-HP Pitié-Salpêtrière, ER1 DETIV, University Paris VI Pierre et Marie Curie, France; Yonsei University College of Medicine, Republic of Korea

## Abstract

**Background:**

A program, supported by the GEMHEP (Groupe d'étude Moléculaire des Hépatites), was established in 2007 in the sanitary district of Tokombéré, to prevent perinatal transmission of hepatitis B virus (HBV). It comprises screening for HBV surface antigen (HBsAg) in all pregnant women and vaccinating the newborn if tests are positive.

**Methods/Principal Findings:**

1276 women were enrolled in the study after providing informed consent. Demographic data and blood samples were available for 1267 of the enrolled patients. HBsAg was determined locally using a rapid test (Vikia HBsAg, Biomerieux). Tests for HBV and HDV virological markers (HBeAg, anti-HDV antibodies (Ab), HBV-DNA, HDV-RNA, HBV and HDV genotypes) were performed on the confirmed HBsAg-positive samples in the virology unit of the Angers University Hospital (France). HBsAg was found in 259 of the 1267 pregnant women (20.4%) between January 2009 and April 2010, of whom 59 were HBeAg-positive (22.7%) with high levels of HBV-DNA. Anti-HDV Ab were found in 19 (7.3%) of the HBsAg-positive women. The prevalence rates of HBsAg and HDV were not age-dependent whereas HBeAg carriers were statistically younger than non carriers. Basal core promoter (BCP) and precore (PC) mutations and genotypes were determined by sequencing. Of 120 amplified sequences, 119 belonged to HBV genotype E (HBV/E) and the 9 HDV strains belonged to HDV clade 1. In the PC region, 83/228 patients (36.4%) harbored a G1896A mutant or mixed phenotype virus. In the BCP region, the double mutation A1762T/G1764A and the G1757A substitution were detected respectively in 26/228 patients (11.4%) and 189/228 patients (82.8%).

**Conclusions:**

Our results confirm the high prevalence and low molecular diversity of HBV in Far Northern Cameroon; more than 20% of the infected women were highly viremic, suggesting a high rate of HBV perinatal transmission and supporting the WHO recommendation to vaccinate at birth against hepatitis B.

## Introduction

According to the World Health Organization (WHO), more than 350 million people are chronic carriers of hepatitis B virus (HBV)[1,2]. HBV chronic infection results in 500,000 to 700,000 deaths per year caused by chronic hepatitis, cirrhosis and hepatocellular carcinoma. HBV is the 10^th^ leading cause of death worldwide [3-5]. Africa is reported to have the highest overall prevalence rate, although there are substantial regional variations ranging from less than 7% to more than 20% [6-9]. In general, the prevalence of HBV surface antigen (HBsAg) increases with age. The earlier the infection occurs in life, the higher the probability of becoming a chronic carrier increases [8,10-12]. Currently, eight genomic groups of HBV are recognized, designated as genotypes A to H. Of these, A and E are considered to be major genotypes and are predominant in central, south, and west Africa [13-16]. Hepatitis delta virus (HDV) is also highly endemic in several African countries [17,18]. Extensive analyses of HDV sequences have illustrated a wide genetic diversity grouped into eight clades [19,20]. However, little information is available on the origin, circulation and genetic diversity of both HBV and HDV infections, especially in Central Africa.

More efficacious treatments, mass immunization programs, and safe injection techniques are essential for eliminating HBV infection and reducing global HBV-related morbidity and mortality. Safe and effective vaccines against HBV infection have been available since 1982. The implementation of mass immunization programs, which have been recommended by WHO since 1991, has dramatically decreased the incidence of HBV infection among infants, children, and adolescents in many countries. However, not all countries have adopted these recommendations and many people infected with HBV prior to the implementation of immunization programs are still alive. In infants born to HBeAg-positive mothers, the reported post-vaccination HBsAg carrier rate ranges from 7.4% to 17.3%, depending on the different types of vaccines, injection schedules, and age of assessment [21]. 

A program was established in 2007 in the sanitary district of Tokombéré, a rural district located in Far Northern Cameroon, to evaluate and prevent mother-to-child transmission of HBV. The program is supported by the GEMHEP (Groupe d'Étude Moléculaire des Hépatites). It involves screening for HBV surface antigen (HBsAg) in all pregnant women and vaccinating newborns when the tests are positive.

The objectives of our study were to establish a more precise measure of the high prevalence of HBV and HDV infection and to analyze HBV genomic diversity in both the polymerase and PC region in this poorly documented area of Cameroon.

## Materials and Methods

### Patient samples

Since 2005, the GlaxoSmithKline foundation has been supporting a program initially dedicated to the prevention of mother-to-child transmission (PMTCT) of HIV. The reach of the program was broadened in 2007 with a new focus on strengthening healthcare both for mothers and for children less than five years of age. Within this setting and with the support of the GEMHEP, a new PMTCT program for HBV was established. We screened all pregnant women for HIV and HBsAg simultaneously. Our project was approved by the Ethics Committee at the University Hospital of Angers, France (Agreement #2013-49). Our data protection has been registered with the CNIL as statement number 1684412. The patients were informed that the samples could be used for research purposes. Only the pregnant women who provided informed consent were included. From January 2009 to April 2010, we collected 1276 sera from consecutive women who had no clinical or biological signs of severe hepatitis. Then, we proposed immunization for the newborns of HBsAg-positive mothers. Immunization comprised HBV monovalent vaccine at birth plus completion of the primary series at 6, 10 and 14 weeks of the Expanded Programme on Immunization with hepatitis B component (EPI-HB) recommended by WHO. 

### Serological testing

Blood samples (5 mL) were collected in the 8 sanitary formations (SF) of the Tokombéré district during the first prenatal consultation after HIV counseling. Initial HIV and HBV tests were performed in the Tokombéré hospital laboratory. HBsAg was detected by a rapid immunochromatographic test (VIKIA HBs Ag, Biomerieux, Marcy l'Etoile, France).

Sera were then stored at -20C° and sent to the virology laboratory of the Angers for complementary serological and molecular tests. The sera were tested with a commercial immunoassay test (ETI-MAK-4, Diasorin, Antony, France) and HBsAg-positive samples were also confirmed by neutralization (Diasorin, Antony, France). In all HBsAg-positive subjects, HBeAg and hepatitis Delta antibodies (anti-HDV Ab) were searched using two ELISA tests, respectively ETI-EBK-PLUS and ETI-ABDELTAK-2 (Diasorin, Antony, France). Anti-HDV IgM was determined in the anti-HDV Ab positive samples (ETI-DELTA-IGMK-2, Diasorin, Antony, France).

### Nucleic Acid extraction

Nucleic acid extractions were performed using the Nuclisens EasyMAG Platform (Biomerieux) according to the manufacturer’s instructions, from 200 µL of serum. HBV DNA and HDV RNA were eluted in 50 µL of elution buffer. Purified nucleic acid was used for HBV DNA and HDV RNA detection and genotyping.

### HBV DNA detection

For all patients, the full length HBV genome was amplified with a qualitative PCR using primers HBV T1 (forward, 5'-cca cca ctt tcc acc aaa ct-3') and HBV T2 (reverse, 5'-gta ggc tgc ctt cct gac tg-3') designed in the Angers. 10 µL of extracted DNA was used for PCR in a 50 µL reaction volume containing colorless GoTaq Flexi buffer 1X, 1.5 mM MgCl_2,_ 100 µM dNTP, 5 U GoTaq Flexi DNA polymerase (Promega) and 300 nM of each primer. Thermal profile amplification in the Verity thermocycler (Life technologies) was: pre-heating at 94°C for 5 minutes, 40 cycles including denaturation at 94°C for 1 minute, annealing at 58°C for 1 minute, and extension at 72°C for 4 minutes, followed by 10 minutes at 72°C. PCR products covering the full HBV genome (3162 bp) were used as templates for two nested PCR.

For polymerase region (P) amplification (809 bp), 5 µL of full length HBV amplicons were mixed in a 50 µL reaction volume containing GoTaq Flexi buffer 1X, 1.5mM MgCl_2,_ 200 µM dNTP, 2.5 U GoTaq Flexi DNA polymerase (Promega) and 200 nM of each primer POL3 (forward, 5'-gac tcg tgg tgg act tct ctc a-3') and POL4 (reverse, 5'-ggc att aaa gca gga tat cca cat tg-3') [22] as previously described. Thermal profile amplification was: pre-heating at 94°C for 5 minutes, 30 cycles including denaturation at 94°C for 40 seconds, annealing at 55°C for 1 minute, and extension at 72°C for 1 minute, followed by 10 minutes at 72°C. PCR products of polymerase gene were controlled by electrophoresis. The result of electrophoresis was used to identify positive and negative samples for HBV DNA detection. Based on our experience, only samples with a viral load >3 log IU/mL were detectable with this in-house PCR method. Positive samples were also used to determine HBV genotype by sequencing.

For PC region amplification (363 bp), 10 µL of full length HBV PCR products were mixed in a 90 µL reaction volume containing GoTaq Flexi buffer 1X, 1.5 mM MgCl_2,_ 200 µM dNTP, 2.5 U GoTaq Flexi DNA polymerase (Promega) and 400 nM of each primer PC3 (forward, 5'-TGTCAACGACCGACCTTGAG-3') and PC4 (reverse, 5'- GCAATGCTCAGGAGACTCTAAGGC- 3') [23] as previously described. Thermal profile amplification was: pre-heating at 94°C for 5 minutes, 30 cycles including denaturation at 94°C for 40 seconds, annealing at 55°C for 1 minute, and extension at 68°C for 1 minute, followed by 10 minutes at 72°C. PCR products of PC gene were controlled by electrophoresis and used to determine PC and BCP phenotypes by sequencing.

For 72 consecutive patients, serum HBV DNA levels were determined with the Abbott Real-Time HBV PCR assay (Abbott) on the M2000sp-M2000rt platform. Due to the small volumes, sera were diluted to a final concentration of 1/5 (v/v) in Abbott RealTime negative HBV control. The lower detection limit was also increased from 1 to 1.7 log IU/mL to take the dilution into account.

### HDV RNA detection

HDV-RNA was detected by PCR using primers *900s* and *1280as*, which encompass a region conserved in all HDV genotypes, i.e., the “R0 region” (400 nt) covering the 3’end of the HDV gene as previously described [24]. PCR products of the R0 region were controlled by electrophoresis and used to determine HDV genotype by sequencing.

### Sequence analysis of HBV and HDV strains

HBV PCR products (covering the P and BCP/PC regions) and HDV PCR products were purified using Nucleospin Extract II columns (Machery Nagel) and then sequenced bi-directionally using the BigDye Terminator v3.1 kit (Life Technologies). After purification, sequences of amplified nucleic acids were determined using an ABI 3130 xl sequencer (Life Technologies) and the nucleotide sequences analyzed with SeqScape® v2.6 software (Life Technologies). Nucleotide sequences of the P region were compared with reference strains representing each of the genotypes A-H, obtained from GenBank. Genotyping of HBV was then determined by blast analysis, and full genome sequences representing the eight HBV genotypes were used as references.

Phylogenic analyses in both the polymerase gene and the BCP and PC gene were performed to assess the genomic diversity of the strains. 

Sequence changes of the PC and BCP regions were used to determine the wild/mixed/mutated PC and BCP phenotypes. In the PC region, two nucleotide changes were analyzed: the presence of a point mutation from G to A at nucleotide 1896 (G1896A), which signals the mutant PC phenotype; and the change C to T at position 1858, which defines the C1858T mutation. In the BCP region, the two-nucleotide substitutions, A-T at nucleotide 1762 and G-A at nucleotide 1764 (A1762T/G1764A), signal the double mutant BCP phenotype. Another mutation was investigated at the 1757 nucleotide position to detect change G^1757^ to A^1757^. Mixed phenotype was defined as the simultaneous presence of both wild and mutated phenotypes.

Genotyping of HDV was determined by blast analysis, and full genome sequences representing the eight HDV genotypes were used as references.

### Statistical analysis

Quantitative variables were compared using Student t test. Qualitative variables were compared by the chi square test or the bilateral Fisher's exact test when the expected value of at least one cell in a contingency table was below 5. A p value of 0.05 or less was considered significant. To assess risk factors, as our study was cross-sectional, we computed risk ratios (i.e., ratio of prevalence in exposed and non-exposed women) and their 95% confidence intervals rather than odds ratios. HBV viral load was expressed as mean ± standard deviation (± SD). All statistical calculations were performed using SPSS software (IBM Corporation, Armonk, NY, USA).

## Results

### HBV and HDV serological screening

None of the 1276 pregnant women had been vaccinated against HBV or had received antiviral treatment. Demographic data and blood samples were available for 1267 of the enrolled patients.

HBsAg was detected in 259 of 1267 sera (20.4%) by the VIKIA HBsAg test. Negative and positive results for HBsAg were confirmed by EIA and neutralization [25,26]. HBeAg was found in 59 of the 259 HBsAg-positive pregnant women (22.7%). Eight pregnant women were anti-HIVAb-positive. Nineteen of the 259 (7.3 %) HBsAg-positive pregnant women were anti-HDV antibody (Ac) positive with anti-HDV IgM in 10/19 (53%) cases.

No difference in age was observed for HBsAg carriers versus non carriers (24.5 years versus 24.3 years; p=0.73) or HDV Ab carriers versus non carriers (26 years versus 24.3 years; p=0.3). Conversely, HBeAg carriers were statistically younger than non carriers (22.9 years versus 24.95 years; p=0.041, 95% confidence interval for the difference of means: 0.08-3.95 years). Tables 1, 2 and 3 summarize the results of the HBsAg, HBeAg and HDV prevalence according to age range (quartiles).

**Table 1:** Prevalence of HBsAg by age group in 1276 pregnant women in the district of Tokombéré.

Variable	HBs Ag		
	n positive/n tested (%)	RR (95% CI)*	P value
Age range (yr)			
< 20	74/335 (22.1%)	1	
20–23	60/330 (18.2%)	0.95 (0.88-1.03)	0.21
24–28	48/278 (17.3%)	0.94 (0.87-1.02)	0.14
> 28	76/324 (23.5%)	1.02 (0.94-1.11)	0.68
All	258/1267 (20.4%)		

* risk ratio and 95% confidence interval

**Table 2:** Prevalence of HBeAg by age group in 1276 pregnant women in the district of Tokombéré.

Variable	HBe Ag		
	n positive/n tested (%)	RR (95% CI)*	P value
Age range (yr)			
< 20	23/74 (31.1%)	1	
20–23	10/60 (16.7%)	0.83 (0.68-1)	0.054
24–28	13/48 (27.1%)	0.95 (0.74-1.2)	0.64
> 28	11/77 (14.3%)	0.80 (0.67-0.96)	0.014
All	57/259 (22%)		

* risk ratio and 95% confidence interval

**Table 3:** Prevalence of HDV total antibodies by age group in 1276 pregnant women in the district of Tokombéré.

Variable	HDV Ab		
	n positive/n tested (%)	RR (95% CI)*	P value
Age range (yr)			
< 20	3/74 (4.1%)	1	
20–23	4/60 (6.7%)	1.03 (0.95-1.12)	0.7^‡^
24–28	5/47 (10.6%)	1.07 (0.97-1.2)	0.26^‡^
> 28	7/76 (9.2%)	1.06 (0.97-1.15)	0.33^‡^
All	19/257 (7.4%)		

* risk ratio and 95% confidence interval

‡ bilateral Fisher's exact test

### HBV DNA detection and quantification

**Figure 1:**
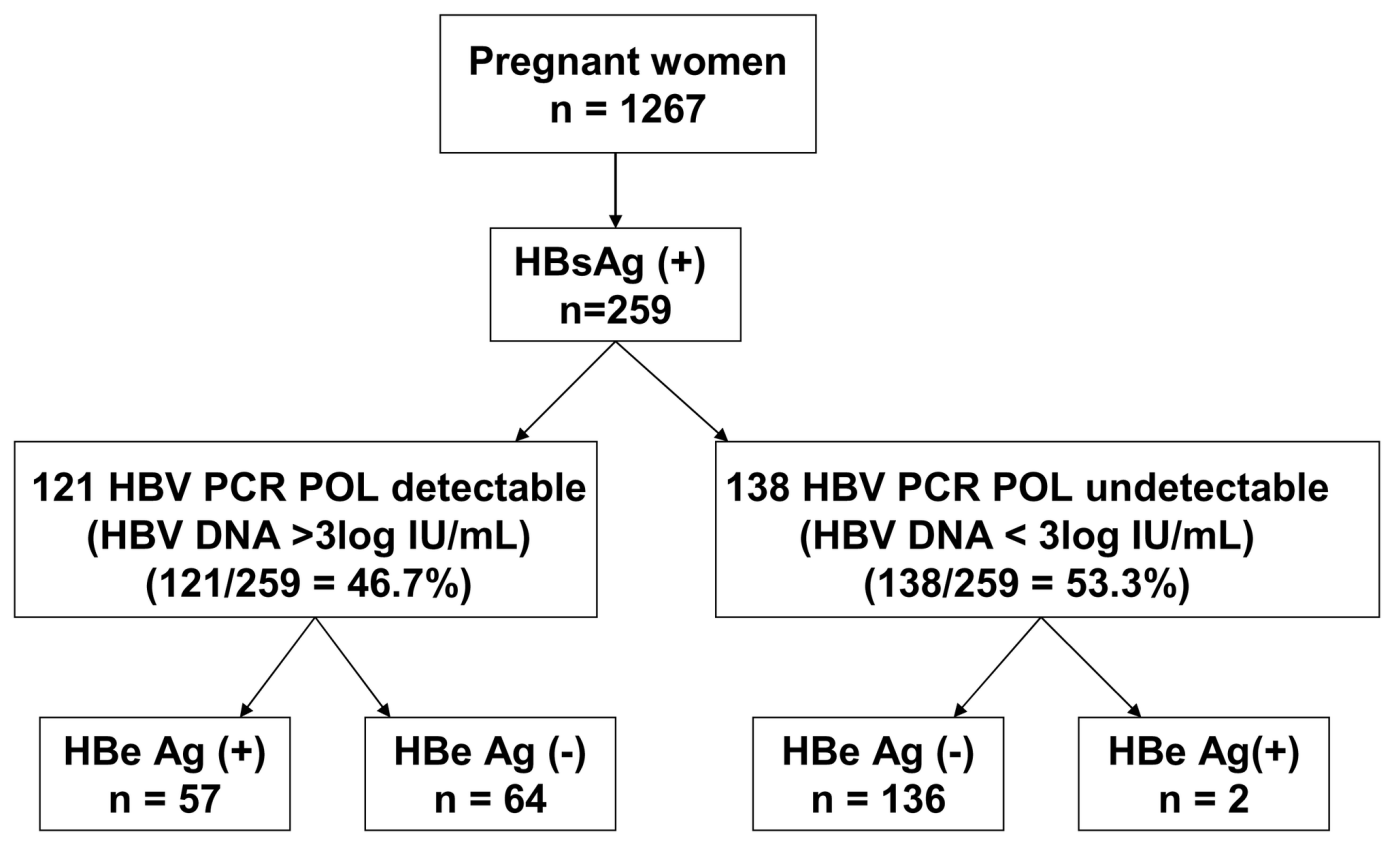
Results of the polymerase gene HBV amplification. The HBV DNA was amplified using a qualitative PCR with a threshold at 3 log UI/mL and the results were correlated with the HBeAg profile in HBsAg-positive pregnant women.

As shown in Figure 1, HBV DNA was detected in 121 of the 259 HBsAg-positive samples (47.1%) by qualitative PCR with a sensitivity ~3 log IU/mL; 57 of these pregnant women (48%) were HBeAg-positive. Thus, in 138 pregnant women (53.3%), HBV DNA was undetectable, 98.5% (136/138) being HBeAg-negative and two HBeAg-positive; interestingly, both of these were co-infected with HDV (HBV DNA levels were <1.7 and 1.97 log IU/mL).

**Figure 2:**
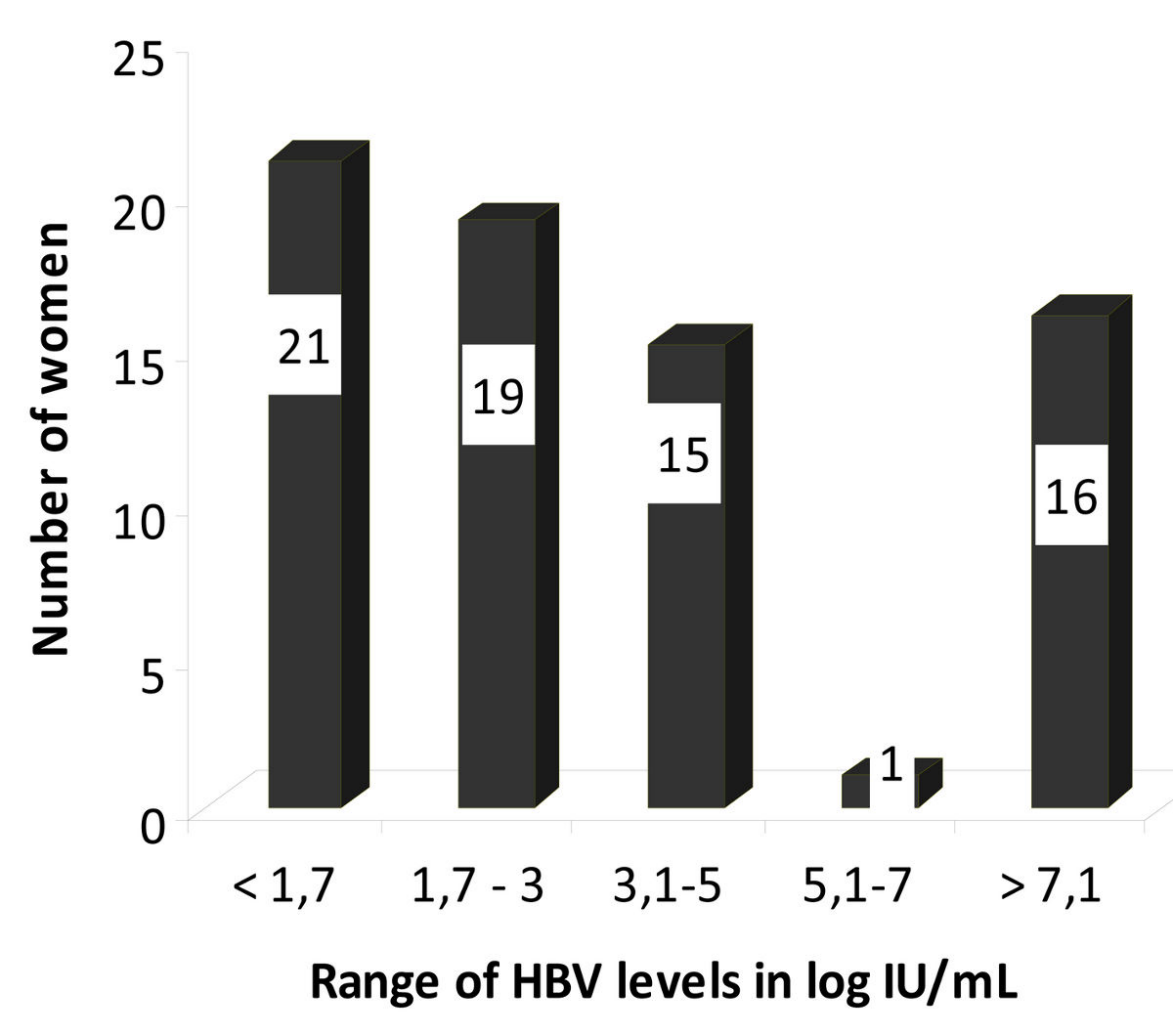
HBV DNA quantification. Serum HBV DNA levels were quantified in 72 consecutive samples with the Abbott Real Time HBV PCR (threshold at 1.7 log IU/mL). HBV target was not detected in 21 patients; viral load was detected in 51 patients, 16 of whom had a viral load greater than 7 log IU/mL.

**Figure 3:**
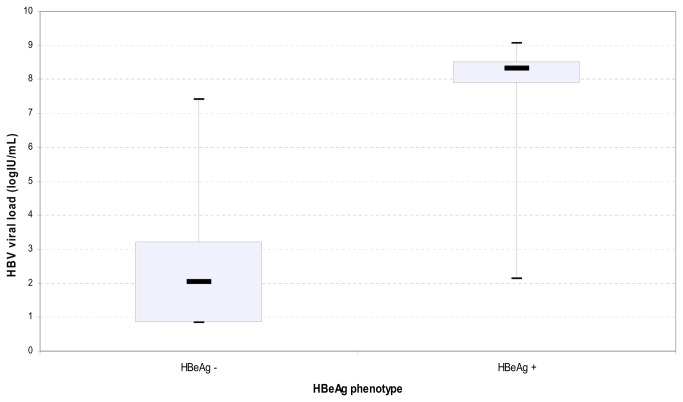
Plots of Log IU/mL HBV-DNA according to the HBe status. Quantification of HBV DNA using the Abbott Real Time HBV PCR: the median of the log IU/mL HBV viral load (solid black bar in the figure) was significantly higher in HBeAg-positive pregnant women than HBeAg-negative ones (8.33 log IU/mL versus 2.05 log IU/mL respectively).

Serum HBV DNA levels were quantified in 72 consecutive samples (qualitative detection of HBV DNA was negative in 43/72 and positive in 29/72 samples). The mean of the viral HBV DNA was 3.44 log IU/mL using the Abbott Real Time HBV PCR, 21 pregnant women did not have a HBV target detected and 51 had a detectable viral load. All women with an undetectable viral load were HBeAg-negative. Among the 51 pregnant women with detectable viral loads, most had an HBV DNA level inferior to 3 log IU/mL. However the viral load was greater than 7 log IU/mL in 16 women (22.2%) (Figure 2). Figure 3 illustrates the repartition of HBV viral load according to HBeAg phenotype. As expected, HBeAg-positive pregnant women had an HBV viral load significantly higher than HBeAg-negative ones: 7.47 (± 2.09) log IU/mL versus 2.2 (± 1.48) log IU/mL (p <0.001).

### HBV and HDV genotyping

Of the 120 samples we were able to amplify in the polymerase gene, 99% (119/120) belonged to genotype E; the one exception was genotype D. Phylogenic analyses both in the polymerase gene and the BCP and PC gene showed that the strains were slightly different and closely related to other strains of African origin, notably the strains we have described in Niger and Mauritania [27,28]. As for HDV, HDV RNA was detected by PCR in 9/19 (47%) cases, all being anti-HDV IgM positive. The 9 HDV strains belonged to HDV clade 1.

### Characterization of BCP/PC phenotypes

PC/BCP sequences were characterized by direct sequencing in 228 samples: 59 HBeAg positive and 169 HBeAg negative. In the position 1896, wild (1896G) mixed (1896G and A) and mutated (1896A) phenotypes were observed in 145/228 (63.5%), 34/228 (14.9%) and 49/228 (21.4%) women respectively (Table 4). The wild PC phenotype was the dominant detected viral population regardless of the HBeAg profile: 94.9% (56/59) and 52.6% (89/169) in HBeAg-positive and -negative patients respectively (p = 0.01). The frequency of mutant or mixed PC populations was higher in HBeAg-negative (80/169) than in HBeAg-positive (3/59) pregnant women (47% versus 5%, p<0.05). Patients who were infected with a mutant PC virus had lower mean HBV DNA viral load than pregnant women who harbored a wild PC virus (3.11 ± 1.66 logIU/mL versus 5.89 ± 2.72 logIU/mL, p < 0.05, data not shown). The mixed/mutated BCP phenotype was detected in 61/228 patients (26.7%); there was no significant difference in the mutant BCP phenotype distribution between the HBeAg-negative and HBeAg-positive pregnant women: 13/59 (22%) and 48/169 (28.4%) respectively (p=0.8). The C1858T and G1757A substitutions were found in 226/228 (99.1%) and 189/228 patients (82.8%) respectively.

**Table 4:** Profile of BCP and PC mutations according to HBeAg status.

			BCP/PC phenotypes		
		Wild Number/Total (%)	Mixed Number /Total (%)		Mutated number/Total (%)
G1896A	HBe (+) Ag	56/59 (94.9%)	2/59 (3.3%)		1/59 (2%)
	HBe (-) Ag	89/169 (52.6%)	32/169 (19%)		48/169 (28%)
	**Total**	**145/228 (63.5%)**	**34/228 (14.9%)**		**49/228 (21.4%)**
G1757A	HBe (+) Ag	3/59 (5%)	0/59 (0%)		56/59 (95%)
	HBe (-) Ag	12/169 (7%)	24/169 (14%)		133/169 (79%)
	**Total**	**15/228 (6.5%)**	**24/228 (10.5%)**		**189/228 (82.8%)**
A1762T/G1764A	HBe (+) Ag	46/59 (77.9%)	5/59 (8.4%)		8/59 (13.5%)
	HBe (-) Ag	121/169 (72%)	30/169 (18%)		18/169 (11%)
	**Total**	**167/228 (73.2%)**	**35/228 (15.3%)**		**26/228 (11.4%)**
C1858T	HBe (+) Ag	1/59 (1.6%)	0/59 (0%)		58/59 (98.3%)
	HBe (-) Ag	0/169 (0%)	3/169 (2%)		166/169 (98.2%)
	**Total**	**1/228 (0.004%)**	**3/228 (1.3%)**		**226/228 (99.1%)**

## Discussion

In the present study, we focused on estimating the prevalence of HBV and HDV in pregnant women in the rural district of Tokombéré in Far Northern Cameroon and exploring virus genome diversity. The population of Tokombéré is currently estimated at 130 000 inhabitants with an average density of 260 inhabitants per km^2^. The prevalence of HBsAg in Cameroon is usually estimated to be between 5 and 10%. The HBsAg prevalence of 20.4% in our study is thus surprisingly high. As concerns other studies conducted in Cameroon, we found only one done recently in pregnant women; it reported, in the Yaoundé area, a prevalence of HBsAg of 7.85% with a very low proportion of HBeAg [29]. Still in Cameroon, the prevalence of HBV infection was assessed at 11.8% in three pygmy groups in 2011 [30] and HBsAg prevalence was reported to be 10.7% in Yaoundé blood donors in 2003 [31]. Our high HBsAg prevalence remains coherent with published data at the scale of central sub-Saharan Africa. Variable endemicity data are reported in other countries: 6.5% in Zambia, 9.5% in Gabon, 10.9 % in Mauritania, 12.6% in Ghana, 13.8% in Senegal, 15.5% in Mali and 25% in Zimbabwe [32-37]. Furthermore, HBV rate also differs intranationally in some countries with areas of high endemicity. In Nigeria, for example, studies reported varying seroprevalence rates of HBsAg seropositivity, i.e., 26.0%, 15.1%, 13.2% and 12.6% according to location [38]. The Tokombéré district is located in a part of Cameroon that is pinched between the politically unstable countries of Chad and Nigeria, both presenting a significant rate of emigration and seemingly high HBsAg prevalence (7-30%) [39]. This may help explain the high rate we found. Another explanation may be the nature of the district, rural and mountainous, making access to health facilities difficult and resulting in home deliveries with a higher risk of perinatal contamination.

Concerning HBeAg prevalence, we found a higher HBeAg prevalence than described by Kfutwah et al. In that study, including 650 pregnant women with a mean age of 26.2 years, none of the HBeAg of the 45 HBsAg-positive samples were reactive [29]. However, Jordis et al reported that the highest HBeAg prevalence (over 50%) was found in 0 to 9 year-old girls. At the reproductive age, HBeAg prevalence was 20-50% [40]. Interestingly, the authors demonstrated that HBeAg prevalence decreased between 1990 and 2005 overall, but that the reduction occurred to a lesser extent in the reproductive age-group of 20-39 year old females. Their systematic literature review showed that, in central sub-Saharan Africa, the highest HBeAg positivity rate (54.86%) was observed in the 0-9 age group. The rate then decreased successively: 40.20% in the 10-19 age group, 29.98% in the 20-29 age group and 22.70% in the 30-39 age-group. Our findings correlated well with those of Jordis et al: the highest HBeAg prevalence we observed was in women <20 years old but it declined thereafter in the others groups, with overall prevalence at 22%.

In our study, 119 of the 120 amplified sequences belonged to HBV genotype E (HBV/E). Inter-sample contaminations were avoided by the phylogenic analysis showing minor nucleotide modification in all strains. In Africa, the spread and evolution of HBV genotypes remain poorly understood, but genotype D seems to be the most prevalent in the north. Genotype A is dominant in the southern and eastern Africa regions while HBV/E predominates throughout a vast region ranging from Senegal to Namibia, then eastward to the Central African Republic. HBV/E is thus the most prevalent genotype found in the countries bordering on or close to Cameroon (e.g., Central African Republic, Democratic Republic of the Congo, Benin, Togo, and Nigeria). In Cameroon, HBV/A and/or HBV/E have both been described, depending on the cohorts studied [41-43]. Co-circulation, recombination and a high number of mixed infections have been detected because of this dual genotypic presence, but no evidence whatsoever of this was detected in the population from the Tokombéré district. A unique genotype, combined with the low genetic diversity of HBV/E, supports the hypothesis of an endemic infection in this area.

Concerning the HBV heterogeneity in the PC region, the most frequently reported mutation is G1896A, which pairs with a T in nucleotide 1858. This base-pairing increases the stability of the stem-loop structure of the encapsidation signal, a critical factor for viral replication; thus non-A genotypes favor the emergence and selection of 1896 PC mutants. Our results confirm those of previous reports indicating a G1896A mutation prevalence in African pregnant women inferior to 50% [33,42]. In Guinea, a similar G1896A mutation prevalence (21%) was described in blood donors infected with HBV/E [44]. In our population, the majority of PC sequences (98%) harbored the C1858T mutation. Many studies have demonstrated the high frequency of this mutation in HBV/E [44,45] as well as in genotypes B-D. In our study, it was surprising to note the low prevalence of the G1896A mutation (21.4%) with the high percentage of the C1858T mutation (99.1%) detected in HBV/E, whereas these two point mutations are usually associated. The low frequency of the PC mutation could be explained by the high prevalence of the G1757A mutation in our population. In the literature, there are few data on the mutation at nucleotide 1757, but it seems to be more prevalent in genotypes D and E and to affect negatively the emergence of BCP mutations [45-47]. A recent paper demonstrated that the G1757A substitution is associated with protection against advanced liver disease (*P* = 0.001).

Concerning the core promoter mutations, 11.3% of the pregnant women harbored strains with the double mutation T1762/A1764. Similar results for BCP mutants have been reported in Ghana [33] and the Central African Republic [48]. Thus, more than half of the pregnant women were infected with viral strains with one or two mutations in nucleotides at positions 1762, 1764 or 1896. A unique triple mutant T1762/A1764/A1896 was found in 11.4% of the strains. 

The impact of mutations in the HBV PC and BCP regions on the course of chronic liver disease is not well established. At the time of inclusion, most of the women were clinically asymptotic, but few biochemical (ALAT) or fibrosis markers were available. However, it could be considered that HBsAg-positive pregnant women with high levels of HBV DNA are in the immune tolerant phase of infection and the others are inactive carriers. The low prevalence of the PC/BCP mutants in conjunction with the high percentage of the G1757A mutation might influence the clinical characteristics of HBV/E. It seems necessary to complete these data with a clinical study aimed at investigating the prevalence of the PC/BCP mutated viruses in HBV/E-infected subjects. 

We also found a HDV prevalence of about 7% in this relatively young asymptotic population. This is substantially lower than the prevalence of 6/17 found in the Tokombéré medical staff [25] and quite lower than the 20% prevalence found in pregnant women in Niger or in Mauritania in recent papers [27,28].

## Conclusions

The high prevalence of HBV infection in pregnant women in the Far Northern Region of Cameroon, together with the relatively high proportion of women with high viral load, may be responsible for:

1-A high risk of vertical transmission of HBV in this population, justifying routine immunization at birth with HBV monovalent vaccine. The EPI-HB started at 6 weeks of age at best may be insufficient to avoid certain mother-to-child contaminations. This justifies the addition of Hepatitis B immunoglobulin (HBIg) or preemptive treatment with nucleos(t)ides analogs, as previously suggested, to immunization at birth [49].

2-A probably higher risk of severe outcome particularly in HBe Ag-positive highly viremic mothers. This may include, in the absence of treatment, decompensated cirrhosis and later hepatocellular carcinoma.

In view of the implementation of vaccination for all newborns in Cameroon, we are now assessing the effectiveness of the targeted HBV MTCTP without HBIg in an at-risk population (HbeAg+ mothers). Should there be a significant vaccination failure rate (transmission > 10%), causes (delay in immunization, mother's viral load, HIV co-infection, HBV escape mutants) will be analyzed to determine the possible need for additional measures (administration of IgHBs at birth or antivirals during pregnancy).
